# Relationship between Prenatal Characteristics and Body Condition and Endocrine Profile in Rabbits

**DOI:** 10.3390/ani11010095

**Published:** 2021-01-06

**Authors:** María-Luz García, Raquel Muelas, María-José Argente, Rosa Peiró

**Affiliations:** 1 Departamento de Tecnología Agroalimentaria, Universidad Miguel Hernández de Elche, Ctra de Beniel km 3.2, 03312 Orihuela, Spain; raquel.muelas@umh.es (R.M.); mj.argente@umh.es (M.-J.A.); 2 Instituto de Conservación y Mejora de la Agrodiversidad Valenciana, Universitat Politècnica de València, P.O. Box 22012, 46071 Valencia, Spain; ropeibar@btc.upv.es

**Keywords:** estradiol, foetuses, leptin, NEFA, progesterone, perirenal fat thickness, placenta, ovulation rate

## Abstract

**Simple Summary:**

Litter size is an essential trait in rabbit production, and it depends on ovulation rate and embryonic and foetal survival. The period between 8 and 18 d of gestation is critical for foetal survival, as the placenta controls foetal nutrition during this period. Ovulation rate and foetal survival at 12 d of gestation are affected by body condition and metabolic and hormonal profile. Higher foetal survival is related to a higher number of vessels arriving at the implantation site, and may be due to higher available space for the foetus.

**Abstract:**

This study evaluated the relationship between prenatal characteristics and body condition and endocrine profile. A total of 25 non-lactating multiparous females were used. Body condition, measured as body weight and perirenal fat thickness, non-esterified fatty acids (NEFA), leptin, progesterone and 17β-estradiol were recorded at mating and 12 d of gestation. Ovulation rate, number of foetuses, ovary and foetal weight, length and weight of uterine horn, available space per foetus and maternal and foetal placental morphometry were recorded at 12 d of gestation. Ovulation rate showed a positive linear relationship with number of foetuses, ovary weight and NEFA. A negative linear relationship between ovulation rate and perirenal fat thickness and leptin was obtained. Ovulation rate was maximum when body weight and 17β-estradiol were 4.4 kg and 22.7 pg/mL, respectively. Foetal weight showed a positive relationship with perirenal fat thickness and a negative relationship with leptin. An increase in progesterone and NEFA concentration was related to a positive linear increase in number of foetuses and in uterine horn weight. Space available per foetus was affected both by the number of vessels that reach the implantation site and by position of the foetus in the uterine horn. In conclusion, body condition during mating and early gestation should be maintained within an optimal range to ensure the best prenatal characteristics. While 17β-estradiol, NEFA and leptin affected the ovulation rate, progesterone and NEFA affected foetal development. The number of vessels that reach the implantation site determines early foetal survival.

## 1. Introduction

A high ovulation rate and high litter size are characteristics of females in the rabbit industry [1]. Ovulation rate reaches higher values than litter size, approximately 20% to 40% of the ovulated ova do not reach gestational term (see review by [2]). Most of these losses occur mainly up to 18 d of gestation [3,4]. The early foetal period, between 8 and 18 d of gestation, is critical for foetal survival, as the placenta controls foetal nutrition during this period [5]. Thus, early foetal survival seems to be associated with the placental development, foetal available space and vascular supply [6,7].

Body condition is traditionally employed to measure the mobilization of fat reserves [8]. Furthermore, an optimal body condition of rabbit females is an important issue considered to improve the effectiveness of reproductive performance [9]. Body reserve status is reflected by changes in some metabolic parameters, such as non-esterified fatty acids (NEFA), and leptin concentrations [10,11]. Briefly, NEFA permits follicle growth, ovulation and development of the *corpus luteum* [12]. Leptin concentration is also related to the reproductive function in rabbit females [12,13] since it is implicated in steroidogenesis [14], ovulation [15], and pregnancy and lactation [16]. Specifically, leptin may act as the critical link between adipose tissue and the reproductive system, indicating whether adequate energy reserves are present for normal reproductive function [17]. Other hormones such as estradiol and progesterone are essential for ovulation and the maintenance of pregnancy [18,19].

A detailed understanding of how ovulation rate and early foetal development and survival is affected by body condition and metabolic and hormonal profile could improve the productivity of rabbit females. Therefore, the objective of this work was to study the relationship between ovulation rate and early foetal characteristics and body condition, NEFA, leptin, 17β -estradiol and progesterone.

## 2. Materials and Methods

### 2.1. Ethics Statement

All experimental procedures involving animals were approved.

### 2.2. Experiment Animals

A total of 25 non-lactating multiparous rabbit females were used. Females belonged to a cross population of two lines selected divergently by uterine capacity [20]. Both lines were derived from the V line [21]. The females were held on the experimental farm at the Universidad Miguel Hernández de Elche (Spain). All animals were reared in individual cages and fed ad libitum with a commercial diet (crude protein, 17.5%; crude fiber, 15.5%; ether extract, 5.4%; ash, 8.1%) during their reproductive life. The photoperiod was 16 h light: 8 h dark.

Females that had finished the forth lactation were mated and blood samples were collected from the central ear artery early in the morning, before feeding, to prevent the effect of feeding [9]. Tubes containing EDTA were used. At 12 d after mating, blood samples were also collected after positive abdominal palpation and then females were euthanized by intravenous administration of sodium thiopental in a dose of 50 mg/kg of body weight (Thiobarbital, B. Braun Medical S.A., Barcelona, Spain). The entire reproductive tract was immediately removed in order to measure reproductive traits. Plasma was obtained after centrifugation at 3000× *g* for 15 min at 4 °C and stored at −20 °C until the metabolite and hormones assays were performed.

### 2.3. Reproductive Traits

Total ovulation rate was estimated as the number of *corpora lutea*. The ovaries were weighted. The implantation sites were considered when foetus, and maternal and foetal placenta, were presented. The number of foetuses in each uterine horn was recorded. Foetuses were classified into live foetuses if normal development was observed, or dead foetuses if they were not developed. The number of blood vessels arriving at the implantation sites and position of each foetus in the uterine horn were counted [6]. The uterine positions were: oviduct (the first foetus nearest the ovarian end), middle (foetus in middle of the uterine horn) and cervix (the last foetus in the uterine horn from the ovarian end). All foetuses with their foetal and maternal placental were removed from the uterine horn and were weighted. The empty uterine horn was weighted and its length was measured. The length of each maternal placenta and the distance between maternal placentas or to the end of the uterine horn were measured. Perimeter and area of foetal and maternal placenta were calculated using the AUTOCAD program.

### 2.4. Metabolite and Hormonal Assays

Non-esterified fatty acid (NEFA, mmol/L) concentrations were analyzed in duplicate, using an in vitro enzymatic colorimetric method (NEFA-C^®^, Wako Chemicals GmbH, Neuss, Germany). NEFA in samples was converted to Acyl-CoA by the action of Acyl-CoA synthetase, under the coexistence with coenzyme A. Obtained Acyl-CoA was oxidized and yielded hydrogen peroxide by the action of Acyl-CoA oxidase. In the presence of peroxidase, the hydrogen peroxide formed yields a blue purple pigment. NEFA concentration was obtained by measuring absorbance of the blue purple colour.

Duplicate aliquots of plasma for the sample tube were assayed. The leptin concentrations were measured by RIA antibody using the multi-species leptin kit (XL-85K, Linco Research Inc.^®^, St. Charles, MO, USA). The detection limit was 1.0 to 50.0 ng/mL Human Equivalents (HE). The 17β-estradiol and progesterone concentrations were assayed using a commercial 125I RIA kit (07-238102 and 07-270102, respectively; ICN Pharmaceuticals Inc.^®^, Diagnostic Division, Costa Mesa, CA, USA). The detection range was 10 to 3000 pg/mL and 0.15 to 80.00 ng/mL, respectively. Intra and inter-assay coefficients of variations were <5% for all hormones.

### 2.5. Body Condition

Body weight and perirenal fat thickness were recorded at mating and 12 d of gestation. Perirenal fat thickness was measured by ultrasound imaging as described by [8], using Justvision 200 SSA-320A Toshiba ultrasound equipment.

### 2.6. Traits

#### 2.6.1. At Mating

Variables measured on each female were body weight, perirenal fat thickness, NEFA, 17β-estradiol, progesterone and leptin.

#### 2.6.2. At 12 d of Gestation

Variables measured on each female were body weight, perirenal fat thickness, NEFA, 17β-estradiol, progesterone, leptin, ovulation rate, number of foetuses and uterine weight and length. Total foetal weight, and total foetal and maternal placenta weight per female were calculated.

Variables measured on each uterine horn were weight and length of uterine horn, ovulation rate and ovary weight per ovary and number of foetus per uterine horn.

Variables measured on each foetus were individual foetal weight, foetal and maternal placenta weight, perimeter and area, and maternal placenta length. The available space per foetus was calculated as the length of its maternal placenta plus one-half the total distance to their two adjacent maternal placentas. For extreme foetuses, available space per foetus was the length from the tip of the uterine horn to the maternal placenta plus the length of its maternal placenta and one-half the distance to adjacent maternal placenta [22].

### 2.7. Statistical Analyses

#### 2.7.1. Differences between Mating and 12 d of Gestation

Body weight, perirenal fat thickness, NEFA, 17β-estradiol, progesterone and leptin were analysed with a model that included fixed effect of moment (mating and 12 d of gestation) and random effect of female. MIXED procedure of SAS was used [23].

#### 2.7.2. Relationship between Traits at Mating

In order to study the relationships between ovulation rate and body weight, perirenal fat thickness, NEFA, 17β-estradiol, progesterone and leptin, the model included the linear and quadratic regression coefficients. If the quadratic regression coefficient was not significant, the linear relationship was tested. In addition, the relationship between ovulation rate and number of foetuses was analyzed. The GLM procedure of SAS was used for these analyses.

The model used for ovulation rate and ovary weight per ovary included the random effect of the female. A MIXED procedure of SAS was used for these analyses including the lineal and quadratic regression coefficients. If the quadratic regression coefficient was not significant, the linear relationship was tested.

#### 2.7.3. Relationship between Traits at 12 d of Gestation

In order to study the relationships between uterine weight and length, total foetal weight and total foetal and maternal placenta weight with perirenal fat thickness, NEFA, 17β-estradiol, progesterone and leptin, the model included the linear and quadratic regression coefficient. If the quadratic regression coefficient was not significant, the linear relationship was tested. The GLM procedure of SAS was used for these analyses.

The model used for traits of the uterine horn included the random effect of the female, and the linear and quadratic regression coefficients. If the quadratic regression coefficient was not significant, the linear relationship was tested. For foetal traits, a random effect of the uterine horn was also included. A MIXED procedure of SAS was used for these analyses.

#### 2.7.4. Blood Supply and Uterine Position

The number of live and dead foetuses according to the number of vessels reaching the implantation site with four levels (1, 2, 3 or more than 3 vessels) and the foetal position in the uterine horn (oviduct, central or cervix) was analysed using Chi-square test.

Traits measured in each foetus were analysed with the model:

Y_ijklm_ =µ + V_i_ + P_j_ + m_ijk_ + h_ijkl_ + b_1_ × NF_ijklm_ + e_ijklm_;

where V_i_ is the number of blood vessels reaching the implantation site of the foetus effect with four levels previously described, P_j_ is the foetal position in the uterine horn effect with three levels previously described, m_ijk_ is the random effect of the female, h_ijkl_ is the random effect of the uterine horn, b_1_ is the regression coefficient of the covariate number of foetuses in each uterine horn (NF_ijklm_) and e_ijklm_ is the residual term.

The MIXED procedure of SAS statistical package was used for the analyses.

## 3. Results

### 3.1. Differences between Mating and 12 d of Gestation

Table 1 shows descriptive statistics of the traits. Body condition differed between mating and 12 d of gestation (Table 2). Body weight and perirenal fat thickness increased 4.6% and 5.7%, respectively and NEFA decreased 20%. Levels of 17β-estradiol and progesterone were similar but leptin increased 22% between mating and 12 d of gestation.

### 3.2. Relationships at Mating

**Figure 1:**
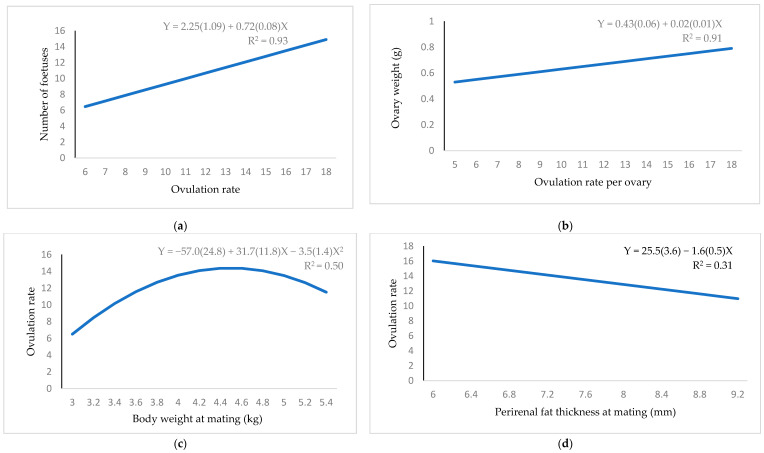
Regression equation (standard error between brackets) at mating for the relationship between: (**a**) ovulation rate and number of foetuses; (**b**) ovulation rate per ovary and its ovary weight; (**c**) body weight and ovulation rate; (**d**) perirenal fat thickness and ovulation rate.

Only significant relationships are shown in the figures. Ovulation rate showed a positive linear relationship with total number of foetuses (Figure 1a). Each extra *corpus luteum* was associated with an increase of 0.72 foetuses. Ovary weight also increased linearly with the ovulation rate (Figure 1b). There was a quadratic relationship between body weight and ovulation rate (Figure 1c). The maximum ovulation rate was reached with 4.4 kg of body weight. There was a linear and negative relationship between ovulation rate and perirenal fat thickness (Figure 1d).

**Figure 2:**
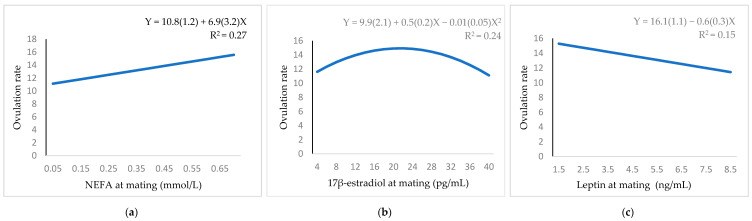
Regression equation (standard error between brackets) at mating for the relationship between ovulation rate: (**a**) NEFA; (**b**) 17β-estradiol; (**c**) leptin.

Figure 2 shows the relationship between ovulation rate with NEFA, 17β-estradiol and leptin. The relationship was positive linear with NEFA (Figure 2a), and negative linear with leptin (Figure 2c). The 17β-estradiol had a significant quadratic relationship (Figure 2b). The equation predicted a maximum of 15.6 *corpora lutea* when the 17β-estradiol was 22.7 pg/mL.

### 3.3. Relationships at 12 d of Gestation

**Figure 3:**
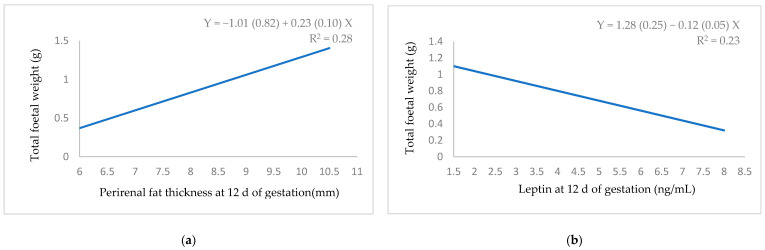

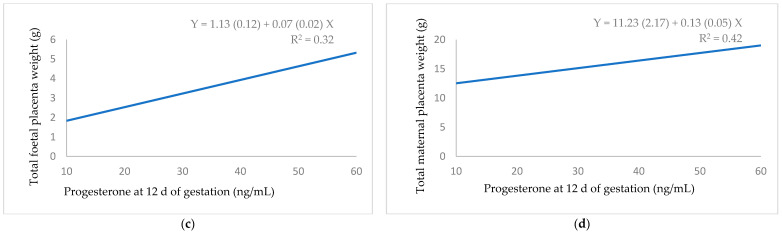
Regression equation (standard error between brackets) at 12 d of gestation for the relationship between: (**a**) perirenal fat thickness and total foetal weight; (**b**) leptin and total foetal weight; (**c**) progesterone and total foetal placenta weight; (**d**) progesterone and total maternal placenta weight.

**Figure 4:**
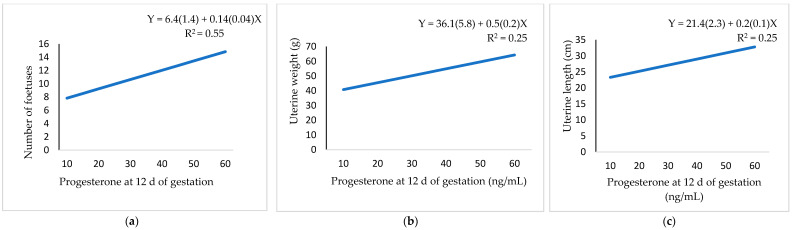
Regression equation (standard error between brackets) at 12 d of gestation for the relationship between progesterone and: (**a**) number of foetuses; (**b**) uterine weight; (**c**) uterine length.

Total foetal weight had a positive relationship with perirenal fat thickness (Figure 3a) and a negative relationship with leptin (Figure 3b). Both the total foetal weight and maternal placenta weight showed a positive linear relationship with progesterone (Figure 3c,d). The number of foetuses (Figure 4a) and weight and length of the uterine horn (Figure 4b,c) showed a positive linear relationship with progesterone.

**Figure 5:**
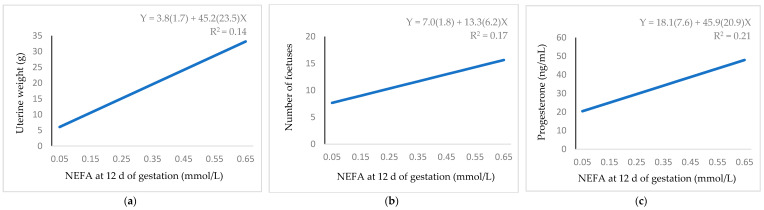
Regression equation (standard error between brackets) at 12 d of gestation for the relationship between NEFA and: (**a**) uterine weight; (**b**) number of foetuses; (**c**) progesterone.

Figure 5 shows the positive linear relationship between NEFA and uterine weight (Figure 5a), number of foetuses (Figure 5b) and progesterone (Figure 5c). Thus, each extra 0.1 mmol/L of NEFA was related to an increase of 4.52 g of uterine weight, 1.33 foetuses and 4.59 ng/mL of progesterone.

### 3.4. Uterine Position, Blood Supply and Foetal Development

**Figure 6:**
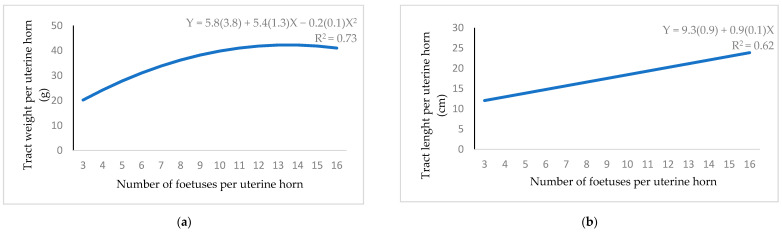
Regression equation (standard error between brackets) at 12 d of gestation for the relationship between number of foetuses per uterine horn and: (**a**) tract weight; (**b**) tract length.

**Figure 7:**
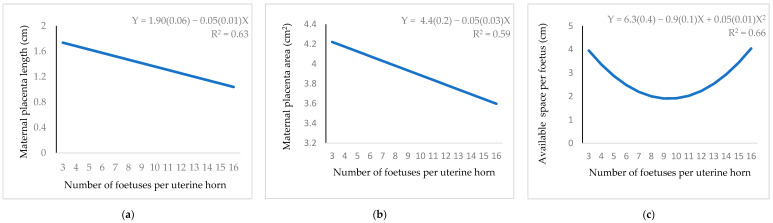
Regression equation (standard error between brackets) at 12 d of gestation for the relationship between number of foetuses per uterine horn and: (**a**) maternal placenta length; (**b**) maternal placenta area; (**c**) available space per foetus.

Number of foetuses showed a quadratic relationship with tract weight (Figure 6a). The maximum weight was 42.5 g when 13.5 foetuses were implanted. A positive linear relationship was seen between number of foetuses and tract length (Figure 6b), but it was a negative linear relationship with maternal placenta length and area (Figure 7a,b, respectively). A convex curve was observed when the relationship between number of foetuses and available space per foetus was studied (Figure 7c). If nine embryos were implanted, the space was minimum (2.2 cm).

Table 3 shows that foetuses with a poor blood supply had a higher probability of death. There were no differences in the percentages of dead foetuses between the different positions within the uterine horn (oviduct, middle or cervix).

Foetal weight was not affected by number of vessels and position in the uterine horn (Table 4). Available space per foetus was lower with one vessel than with more than two vessels. Available space per foetus was lower in the middle of the uterine horn than in the oviduct and cervix.

Foetal placenta weight was higher with three or more vessels reaching the implantation site than with one vessel. The highest foetal placenta perimeter and area were shown when more than three vessels reached the implantation site (4.97 cm and 2.07 cm^2^, respectively). Regarding the position in the uterine horn, foetal placenta weight was 6% higher in the middle than in the oviduct and cervix. Neither the perimeter nor the area was affected by the position.

A maternal placenta with three or more vessels showed higher weight than with less than three vessels. Length of maternal placenta was higher for two or more vessels, and this length was higher in the oviduct than in the middle, but similar in the cervix (Table 5).

## 4. Discussion

### 4.1. Relationships at Mating

Body condition, measured as body weight and perirenal fat thickness, is a common tool for assessing the energy status of rabbit females. Moreover, NEFA is used to measure energy mobilization [9]. While body condition increases from mating to 12 d of gestation, NEFA level decreases. Leptin is also higher during gestation due to the role it plays in foetal development. Therefore, gestation induces a hormonal and metabolic adaptation necessary to fulfil the energy requirements of both females and foetuses [24].

Concentration of 17β-estradiol and progesterone at mating is similar to 12 d of gestation. Similar results were found by Fortun et al. [25]. 17β-estradiol is essential for ovulation and normal luteal function in the pregnant females [18]. A peak of progesterone has been shown at mating with a similar concentration during gestation [26].

### 4.2. Relationships at Mating

Ovulation rate is affected by body condition and metabolic and hormone profile. We have found a quadratic relationship between body weight at mating and ovulation rate. Thus, depleted body weight or being overweight have a negative effect on ovulation rate. A similar relationship was found for sexual receptivity and fertility [27]. Our results demonstrated that a high perirenal fat thickness produces a lower ovulation rate and this could account for the lower litter size at birth found in females with high perirenal fat thickness [28].

The ovulation rate is influenced by levels of NEFA, leptin and 17β-estradiol at mating but it is not affected by progesterone. NEFA acts at the ovarian level by modifying endocrine, paracrine, and autocrine regulation, which permit follicle growth, ovulation and development of the *corpus luteum* [12]. The ovulation rate is positively related to NEFA concentration. A higher number of oocytes would increase the energy demand, which was reflected in the higher NEFA concentration [29] and consequently lower leptin [11]. Leptin plays a dual role in regulating reproduction [30]. On the one hand, a minimum threshold level of leptin is necessary to ensure normal reproduction [31]. On the other hand, elevated leptin levels negatively influence normal ovarian function and oocyte quality [32,33]. Our results confirm the negative relationship between leptin concentration and ovulation rate.

Measurements of 17β-estradiol levels have been used to assess follicular growth [34]. Thus, high levels of 17β-estradiol were related to a high population of antral follicles [35] that would ultimately imply a higher ovulation rate. But the quadratic relationship between both traits would indicate that an excess of 17β-estradiol levels would decrease the ovulation rate.

### 4.3. Relationships at 12 d of Gestation

An optimal body condition of females during gestation improves birth weight and litter uniformity [36]. It seems that perirenal fat thickness plays an important role in early foetal development. While body weight at 12 days of gestation does not show any relationship with the early foetal characteristics, the perirenal fat thickness is positively related to foetal weight. Similar results have been found in pigs [37].

The levels of progesterone and NEFA present a linear and positive relationship with the prenatal characteristics. High progesterone concentrations are necessary for high prenatal survival [38]. Our results would indicate that it could be due to the fact that progesterone increases not only length and weight of uterine horn but also placenta weights. Moreover, a high uterine weight and number of foetuses implies a greater energy expenditure that entails increasing the levels of NEFA in plasma during gestation [11,24]. We have found that leptin decreases proportionally with foetal weight, which is consistent which this NEFA increment.

The 17β-estradiol is essential for normal luteal function in the pregnant females [18] and therefore to maintain the gestation. However, no relationship has been found between 17β-estradiol and the number of foetuses or the uterine and placental characteristics.

### 4.4. Uterine Position, Blood Supply and Foetal Development

Female rabbits have a duplex uterus, i.e., constituted by two separated fully functional uterine horns and cervices opening into a sole vagina. Therefore, embryo inter-horn migration is not possible [39]. This anatomical characteristic implies that foetal growth depends on their number, irrigation and position in the uterine horn [40,41].

The number of foetuses shows a convex curve with uterine horn weight but the curve is concave for space available per foetus. A reduction in the available uterine space, could increase the number of dead foetuses [21]. Each embryo requires a certain minimum space of uterus to attach, survive, and develop, as previously indicated in pigs [42] and rabbits at 25 d of gestation [6]. Maternal placenta length and area are the traits negatively affected by the increase in the number of foetuses.

At 12d of gestation, two or more vessels reaching implantation point guarantee that more than 98% of foetuses survive. Nevertheless, number of vessels is increased to three or more for a 90 or 95% of survival at 18 d of gestation [43]. The higher foetal placenta weight, perimeter and area, and the higher placenta maternal weight and length found in foetuses with a higher number of vessels could increase foetal survival at the middle of gestation. However, foetal weight is the most important parameter to guarantee foetal survival at the end of gestation [6].

The foetuses in the middle of the uterine horn had a lower availability space and lower length of maternal placenta than those near the oviduct or cervix because their littermates flanked them on both sides. However, this condition does not affect their survival. These results were confirmed at 18 d and 25 d of gestation [6,43]. It seems that the degree of irrigation is a more determining factor than the position of the foetus in the uterus to guarantee its survival.

## 5. Conclusions

In conclusion, body condition during mating and early gestation should be maintained within an optimal range to ensure the best prenatal performance. While 17β-estradiol, NEFA and leptin is related to ovulation rate, progesterone, NEFA and leptin levels affect early foetal development. The number of vessels that reach the implantation site determines foetal and placental development and therefore early foetal survival.

## Figures and Tables

**Table 1:** Summary statistics of the traits.

Trait	N	Average	Minimum	Maximum	Standard Deviation
At mating					
Body Weight (Kg)	25	4.10	3.65	5.25	0.41
Perirenal Fat Thickness (mm)	25	7.68	6.04	9.16	0.92
Non-esterified fatty acids (mmol/L)	25	0.34	0.08	0.70	0.15
17β -estradiol (pg/mL)	25	20.01	4.20	39.23	10.73
Progesterone (ng/mL)	25	30.82	9.67	72.25	18.47
Leptin (ng/mL)	25	3.89	1.28	8.75	2.11
At 12 d of gestation					
Body Weight (Kg)	25	4.28	3.94	5.13	0.30
Perirenal Fat Thickness (mm)	25	7.97	6.24	9.95	1.08
Non-esterified fatty acids (mmol/L)	25	0.28	0.11	0.47	0.10
17β -estradiol (pg/mL)	25	22.61	12.24	33.23	5.05
Progesterone (ng/mL)	25	30.31	9.18	62.19	15.14
Leptin (ng/mL)	25	4.48	1.52	8.40	1.68
Traits per female					
Ovulation Rate	25	13.31	8.00	19.00	2.54
Number of Foetuses	25	10.32	3.00	18.00	3.59
Uterine Weight (g)	25	51.25	22.45	69.64	12.98
Uterine Length (cm)	25	27.31	12.70	34.04	5.28
Total Foetal Weight (g)	25	0.85	0.09	2.31	0.58
Total Foetal Placenta Weight (g)	25	3.28	0.43	7.17	1.89
Total Maternal Placenta Weight (g)	25	14.24	2.89	27.07	5.47
Traits per uterine horn					
Ovary Weight (g)	50	0.60	0.36	0.94	0.14
Ovulation Rate	50	6.56	2.00	13.00	2.22
Number of Foetuses	50	5.34	1.00	11.00	2.44
Tract Weight (g)	50	25.36	8.14	47.98	9.14
Tract Lenght (cm)	50	14.24	8.41	21.60	2.79
Traits per foetus					
Foetal Weight (g)	261	0.13	0.02	0.46	0.08
Foetal Placenta Weight (g)	261	0.35	0.03	0.90	0.18
Foetal Placenta Permieter (cm)	261	4.50	1.36	6.53	1.00
Foetal Placenta Area (cm^2^)	261	1.54	0.19	5.78	0.85
Maternal Placenta Weight (g)	261	1.34	0.15	3.10	0.46
Maternal Placenta Perimeter (cm)	261	7.85	3.41	13.15	1.20
Maternal Placenta Area (cm)	261	3.79	1.45	7.16	0.96
Maternal Placenta Length (cm)	261	1.60	0.23	2.26	0.34
Aviable space per foetus (cm)	261	2.82	0.50	10.56	1.16

**Table 2:** Least square means and standard error of body weight, perirenal fat thickness, 17β -estradiol, NEFA, progesterone and leptin at mating and 12 d of gestation.

Trait	Mating	12 d of Gestation
Body Weight (Kg)	4.09 ± 0.04 ^a^	4.28 ± 0.04 ^b^
Perirenal Fat Thickness (mm)	7.68 ± 0.10 ^a^	8.12 ± 0.10 ^b^
Non-esterified fatty acids (mmol/L)	0.35 ± 0.02 ^b^	0.28 ± 0.01 ^a^
17β-estradiol (pg/mL)	19.72 ± 1.61	22.93 ± 1.54
Progesterone (ng/mL)	31.71 ± 3.42	30.69 ± 2.55
Leptin (ng/mL)	3.85 ± 0.11 ^a^	4.69 ± 0.14 ^b^

^a,b^ Different superscripts on the same line differ at *p* < 0.05.

**Table 3:** Percentage of live and dead foetuses according to the number of vessels reaching the implantation site and the position of the foetus in the uterine horn.

Trait	Number of Vessels				Position		
	1	2	3	>3	Oviduct	Middle	Cervix
Live foetuses (%)	83	98	99	98	92	97	98
Dead foetuses (%)	17	2	1	2	8	3	2
χ^2^ = 19.24 *p* = 0.0002					χ^2^ = 3.45 *p* = 0.18		

**Table 4:** Least square means and standard errors of foetal weight, available uterine space per foetus and foetal placenta morphometry according to the number of vessels reaching each implantation site and the position of the foetus in the uterine horn.

Effect	Level	Foetal		Foetal Placenta		
		Weight (g)	Available Space (cm)	Weight (g)	Perimeter (cm)	Area (cm^2^)
Number of vessels	1	0.127 ± 0.02	2.77 ± 0.14 ^a^	0.306 ± 0.03 ^a^	4.30 ± 0.20 ^a^	1.52 ± 0.15 ^a^
	2	0.128 ± 0.01	3.03 ± 0.09 ^ab^	0.355 ± 0.02 ^ab^	4.38 ± 0.11 ^a^	1.48 ± 0.09 ^a^
	3	0.136 ± 0.01	3.16 ± 0.10 ^b^	0.371 ± 0.02 ^b^	4.63 ± 0.13 ^a^	1.50 ± 0.11 ^a^
	>3	0.116 ± 0.01	3.21 ± 0.14 ^b^	0.378 ± 0.03 ^b^	4.97 ± 0.16 ^b^	2.07 ± 0.17 ^b^
Position	Oviduct	0.123 ± 0.01	3.58 ± 0.11 ^b^	0.358 ± 0.03 ^a^	4.58 ± 0.16	1.69 ± 0.14
	Middle	0.127 ± 0.01	2.77 ± 0.07 ^a^	0.339 ± 0.01 ^b^	4.48 ± 0.08	1.59 ± 0.08
	Cervix	0.131 ± 0.01	3.19 ± 0.13 ^b^	0.361 ± 0.03 ^a^	4.66 ± 0.16	1.65 ± 0.15

^a,b^ Rows within the same column with a different superscripts on the same line differ at *p* < 0.05.

**Table 5:** Least square means and standard errors of the maternal placenta morphometry according to the number of vessels reaching each implantation point and the position of the foetus in the uterine horn.

Effect	Level	Placenta			
		Weight (g)	Perimeter (cm)	Area (cm^2^)	Length (cm)
Number of vessels	1	1.26 ± 0.07 ^a^	7.58 ± 0.22	3.79 ± 0.17	1.47 ± 0.05 ^a^
	2	1.29 ± 0.04 ^a^	7.70 ± 0.13	3.68 ± 0.10	1.60 ± 0.03 ^b^
	3	1.42 ± 0.06 ^b^	7.85 ± 0.15	3.77 ± 0.12	1.68 ± 0.04 ^b^
	>3	1.43 ± 0.06 ^b^	8.03 ± 0.20	3.95 ± 0.16	1.66 ± 0.05 ^b^
Position	Oviduct	1.31 ± 0.07	7.68 ± 0.18	3.72 ± 0.15	1.70 ± 0.04 ^a^
	Middle	1.33 ± 0.04	7.91 ± 0.11	3.82 ± 0.08	1.60 ± 0.02 ^b^
	Cervix	1.42 ± 0.07	7.79 ± 0.19	3.86 ± 0.15	1.65 ± 0.04 ^ab^

^a,b^ Rows within the same column with a different superscript indicate significant differences (*p* < 0.05).

## Data Availability

The data presented in this study are available on request from the corresponding author.
